# *Helicobacter pylori *- a seasoned pathogen by any other name

**DOI:** 10.1186/1757-4749-1-24

**Published:** 2009-12-23

**Authors:** Niyaz Ahmed, Shivendra Tenguria, Nishant Nandanwar

**Affiliations:** 1 Pathogen Biology Laboratory, Department of Biotechnology, School of Life Sciences, University of Hyderabad, Hyderabad, India

## Abstract

*Helicobacter pylori *is a well known inhabitant of human stomach which is linked to peptic ulcer disease and gastric adenocarcinoma. It was recently shown in several studies that *H. pylori *can be harnessed as a surrogate marker of human migration and that its population structure and stratification patterns exactly juxtapose to those of *Homo sapiens*. This is enough a testimony to convey that *H. pylori *may have coevolved with their host. Several protective effects of *H. pylori *colonization have been considered as evidence of a presumed symbiotic relationship. Contrary to this assumption is the presence of a strong virulence apparatus within *H. pylori*; why a co-evolved parasite would try inflicting its host with serious infection and even causing cancer? The answer is perhaps embedded in the evolutionary history of both the bacterium and the host. We discuss a hypothetical scenario wherein *H. pylori *may have acquired virulence genes from donors within its environment that varied with change in human history and ecology. The *H. pylori *genomes sequenced to date portray fairly high abundance of such laterally acquired genes which have no assigned functions but could be linked to inflammatory responses or other pathogenic attributes. Therefore, the powerful virulence properties and survival strategies of Helicobacter make it a seasoned pathogen; thus the efforts to portray it as a commensal or a (harmless) 'bacterial parasite' need rethinking.

## Introduction

The human gastric pathogen *Helicobacter pylori *is the most successful colonizer in the stomachs of almost half of the world's population. As a result of this colonization, a majority of infected individuals show histological signs of chronic gastritis; only a small fraction of infected individuals develop *H. pylori*-associated diseases, such as peptic ulcers and, more rarely, gastric adenocarcinomas [1]. Consequently, each year, about half a million patients die from gastric cancer worldwide, making it one of the three major causes of cancer related deaths, leading to considerable socioeconomic costs. As for many other cancers, chemotherapy of gastric adenocarcinoma hardly leads to any improved outcome; however, it is possible that the development of *H. pylori*-associated gastric cancer can be prevented by eradication or abrogation of the infection. The pathology of *Helicobacter *induced chronic gastric inflammation entails a highly coordinated interplay [2] of several virulence factors (encoded mainly by the accessory component of the bacterial genome), although CagA being the most important single virulence determinant that has been investigated extensively for mechanistic and functional evidence to its being cytotoxic and carcinogenic [3-7]. Host genetics and the environment play important roles in imparting susceptibility (or otherwise) towards more serious outcomes of the colonization.

On the other front, *H. pylori *has been projected to have co-evolved with its human host [8], and several protective roles played by the colonizer have been speculated [9,10]; accordingly, low incidences of gastroesophageal reflux disease and childhood diarrhea, and lately asthma, have been suggested to be linked to the presence of *H. pylori *[1,9-11]. Despite the deemed protective roles/associations of the *H. pylori *colonization, the pathogen needs to be carefully monitored especially in developing countries, where widespread drug resistance makes it difficult to be eradicated creating thus a persistent colonizer. In the future, global climate change is likely to impact transmission dynamics of *H. pylori *as there has been an active link of the same with the climate change due possibly to its being transmitted through contaminated water amidst poor community hygiene (at least in the developing countries) and it is shown to be a predisposing factor for the incidence of water-borne diseases such as cholera [12]. Given this, novel intervention strategies are needed at the level of prevention of transmission and therapy. Replicate genome sequences of the pathogen appear promising [13] to understand acquisition and maintenance of virulence in an evolutionary sense; consequently, novel information could emerge to contribute in terms of understanding virulence mechanisms leading to chronic adaptation and survival.

## Co-evolution and acquisition of virulence

Polymorphisms in the genomes of pathogens potentially provide support for the reconstruction of ancestral human population migrations and settlements. This is particularly true for microorganisms that persist lifelong and cause overt chronic diseases. Population genetic structure of such pathogens (that are supposedly co-evolved with humans) juxtaposes to genetic distribution patterns of their host. Human DNA analysis in the recent past has revealed that the farther from Eastern Africa a population is, the more diverse genetically it is (as compared to other human populations) [14]. Comprehensive genetic analysis of *H. pylori *found almost exactly the same dispersal scenario for this pathogen [8]; genetic affinities estimated based on multilocus sequence typing (MLST) of many different sets of *H. pylori *isolates revealed a co-evolutionary pattern [15-17], meaning that population genetic structure of its human host could be similar to the population stratification patterns of the pathogen. Apart from this, genetic analyses incorporating human and bacterial data sets lend support to the idea that *H. pylori *may have migrated from Eastern Africa at almost exactly the same time as early humans, approximately 60,000 years ago [8]. This ultimately conveys that humans and this bacterium have been intimately linked at least for the last 60,000 years. However, the question that has not been answered clearly until now is whether this 60, 000 year old *H. pylori *was as virulent as today's *H. pylori*? In other words, it is not clear if *H. pylori *harbored its virulence genes since the beginning or it acquired them later (from early microorganisms that surrounded humans as a result of gradual change in history and ecology of the early societies through their transition from hunter gatherer's lifestyle to the agrarian type). Also, it is not clearly known which of the deleterious genes were lost from the genome to achieve a peaceful coexistence with the host.

**Figure 1:**
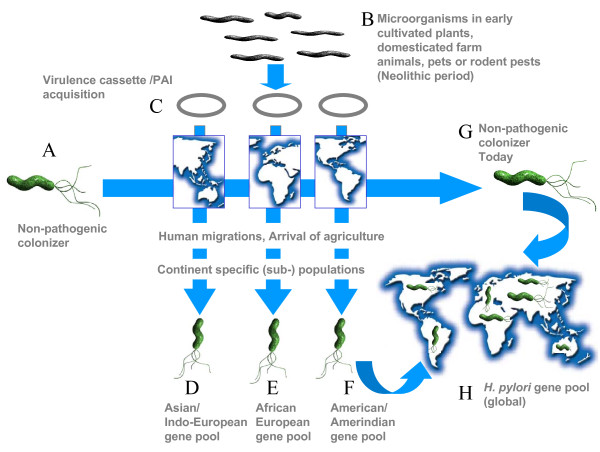
**Genome evolution, global diversification and spread of *H. pylori *(sub-) populations**. Horizontal gene transfer and genome plasticity likely contributed to the evolution of pathogenic variants from non-pathogenic colonizers. Modern *H. pylori *populations thus derived their gene pools from ancestral populations that arose on different continents and can be correlated with different migrations of human populations and other Neolithic events such as arrival of agriculture. The beginning of agriculture and the domestication of farm animals (which seem to have occurred hand in hand but across multiple domestication events in a continent specific manner) suggest a scenario, as depicted here, which can be linked to the acquisition of virulence by *H. pylori*. It can be hypothesized that early bacterial communities originating from crop plants, animals or rodent pests etc. rampant in the vicinity of early human societies may have served as donors of some of the virulence gene cassettes. Such genetic elements may have been acquired by *H. pylori *either bit by bit or en-bloc, at some point of time, through horizontal gene transfer events. There are indirect evidences to this effect in the form of sequence and structural similarities of some of the *H. pylori*'s virulence genes to their homologues in plant pathogens and environmental bacteria. Also, we believe that the extraneous virulence genes may have conferred some survival advantage upon *H. pylori *making them fitter in different human and animal hosts and, as a result, the pathogen may have spread selectively in a geographically compartmentalized manner.

The acquisition of the *cag *pathogenicity island (PAI) has therefore been the subject of debate on its origin and circumstances under which the PAI was imported from a foreign source [16]. Taking into account the comprehensive genetic analyses that have been performed, it is possible to predict a possible evolutionary scenario (Fig. 1) that supports the proposition that the *cag*PAI was acquired by ancestral *H. pylori *populations that arose on different continents before agriculture began in the civilized world. The acquisition of the PAIs might have occurred in *H. pylori *populations quite recently, possibly due to close contact of humans with domesticated animals, crops or rodent pests surrounding them. Such an inter species gene transfer could be explained partly based on the fact that many constituent genes of the *cag*PAI reveal well-established homologies to the type IV systems of *Agrobacterium tumifaciens *[18] and that *cag*A-like sequences have been reported from some *Aeromonas *isolates [19], obtained from environmental samples. Subsequent environmental changes and evolution of the food habits might have led to further continent specific adaptation of *H. pylori*. To date, the genetic structure of *H. pylori *is highly geographically oriented, both with respect to the core and the flexible genome components.

Also, the gain of pathogenicity islands might have augmented the fitness of the organism to infect and spread, thus giving rise to modern populations capable of out competing any residual, native strains. This has in fact been suggested in case of South American (Amerindian) strains of Helicobacter which are gradually disappearing as a result of colonization by more 'aggressive' European strains [16]. Many of the genetic elements supposedly of foreign origin in *H. pylori *have been described to be virulence-linked in a strain specific manner. That means, for some strains, enhanced pathogenic and proinflammatory potentials are imparted by novel elements which may not be universally conserved [20,21].

## Survival tactics, chronicity and disease association - role of strain specific genes

*H. pylori *induced chronic gastritis is a definitive risk factor for the development of gastric cancer. However, it was found that the statuses of some of the chief virulence factors (CagA and VacA) do not always correlate with particular outcomes of infection, as also discussed previosuly [22]. In view of this, it appears that virulence of *H. pylori *is a complex phenotype that need to be seen as a function of bacterial strategies aimed at survival and adaptation. However, it is not clear how the bacterium maintains its niches for almost an entire life span of its host without being cleared. Perhaps, there operate highly orchestrated, biological interactions between the host and the pathogen; the nature of such interactions is not clearly understood. Of late, roles of new virulence determinants are becoming plausible. *H. pylori *harbors up to 45% strain specific genes [23], mostly gained through horizontal gene transfer events [24]. Recently, some of the members of the plasticity region cluster were proposed to be likely involved in promoting proinflammatory capacity of some of the strains [22,25] thus imparting a survival advantage. Our experiments with one such protein, from the plasticity region cluster suggest that some of the members of this cluster encode proinflammatory and/or proapoptotic roles (Alvi *et al*., unpublished data). Most persistent microbes seemingly evolve strategies to avenge innate responses to gain niche and to maintain growth fitness. For example, *H. pylori *traditionally harness its chief virulence factors, CagA and VacA to cause pathology *via *a two pronged approach: 1) downregulate T-cell responses (through the VacA mediated cell cycle arrest) and 2) upregulate mucosal proinflammatory pathways (by CagA). Surprisingly, in our studies, one of the plasticity region cluster protein appeared to be able to perform both the immune stimulatory (macrophage proliferation, secretion of IL8 and TNF-alpha) and immune evasion (apoptosis of activated macrophages) tasks single handedly (unpublished observations). Thus we believe that some of the bacterial proinflammatory proteins [such as JHP0940 [22] and others] are capable of taking up the functions of Vac A/Cag A, especially in the case of the deficiency of the latter and probably function as 'persistence factors' (Alvi *et al*., unpublished data); this however awaits validation using appropriate animal models.

In a recent study [25], 42 isolates of *H. pylori *were profiled to find that 1,319 genes were present in all isolates, while 341 (20.5%) genes were variably present among different isolates. Of the variable genes, 127 (37%) were interspersed within the plasticity region cluster. They observed disease association of such genes and found thirty genes to be significantly associated with nonatrophic gastritis, duodenal ulcer, or gastric cancer, 14 (46.6%) of such putative disease-linked genes were operational from within the plasticity region and the cag PAI (many of the constituent genes of the cag PAI form part of the plasticity region cluster of *H. pylori*). In the observation of Romo-Gonzalez [25], two genes (HP0674 and JHP0940) were absent in all gastric cancer isolates. In our own studies (Tenguria and Ahmed, unpublished), strains representing intestinal metaplasia cases failed to amplify JHP0940 gene (data not shown). It is therefore possible that some of the genes are deleted by the pathogen as the disease progresses through intestinal metaplasia to gastric cancer. However, such observations need functional validation and mechanistic explanation. Nevertheless, the disease-linked genes as discussed above, may be pursued as (putative) biomarkers of the risk for progression of *H. pylori *induced inflammation towards more serious gastroduodenal illnesses such as atrophic gastritis, intestinal metaplasia and gastric adenocarcinoma.

## Association with other enteric infections

In the last two decades, several researchers have predicted mass migrations as a consequence of climate change. They have foreseen millions of people fleeing from rising sea levels, floods, disease outbreaks and drought, leading to serious consequences for both migrants and receiving societies. Many enteric infection outbreaks occur during shortage of drinking water wherein populations are forced to drink from un-conventional or unreliable sources which might be contaminated by sewage and human excreta. Enteric pathogens such as *H. pylori *also co-migrate with their human host. Population dynamics and disease potentials of these pathogens are likely to change with the change in history, geography and ecology of their hosts. *H. pylori *is thus one of the prominent candidates whose epidemiology and evolution within different stationary and migratory communities will be of interest as the impact of climate change as well as change of lifestyle [26] on enteric infection has emerged as a concern in recent years. Chronic *H. pylori *infection has already been described to be a predisposing factor for enteric infections such as cholera [12], which occurs mostly as a result of groundwater contamination - a potential sequel of local or global climate change.

## Replicate *H. pylori *genomes - how many do we need?

Like many other pathogenic bacteria, *H. pylori *is being sequenced to generate replicate, whole genome sequences. Such replicate genomes [13,27], are likely to yield novel, 'back up' functions encoded from within a 'dockyard' of accessory genes called the 'plasticity region cluster' [20]. Previous studies point to such pool of strain specific genes in pathogens such as *H. pylori*, which could be useful in adaptation to a particular host population [21,23-25]. Another important reason to sequence replicate genomes of *H. pylori *entails the need to study chronological evolution within a single host. The nature and extent of genetic rearrangement that the chronically inhabiting pathogens such as *H. pylori *accumulate (across wide timescales) and during colonization of different host niches are not known; the advantages of polymorphisms that impart needed fitness to pathogens or commensals to colonize and inhabit their preferred host (niches) need additional in-depth studies [13]. While some experiments have been conducted to explore chronological strain diversity through multilocus genotyping [28] microarrays [29] and limited sequencing [22], whole genome profiling of such isolates has not been performed. This needs to be done at the earliest, especially, for those strains which are obtained at different intervals and sampled from different sites of individual patients to investigate the occurrence of possible insertions, deletions and substitutions (and mechanisms thereof) including their functional significance related to host adaptation and gain of niche. Apart from this, geographically distinct strains and their multiple representatives could be sequenced to explore local advantages that prevail in certain geographical regions in terms of host adaptation or disease outcome; for example, *H. pylori *infection in the Indian population (despite a very high colonization rate of up to 90%) rarely leads to serious consequences such as gastric cancer in a significant majority of patients who test positive for *H. pylori *infection [26]. Biological co-ordinates of such 'protection', if any, should be studied with the help of bacterial genome sequence data obtained from a number of strains. This appears not a distant possibility given that the next-generation sequencing methods are becoming increasingly affordable. Also, the costs of whole genome sequencing should be low given that the genome sequence is approximately about 1.67 Mb.

## Parasite, commensal or a mutualist?

If we discuss survival advantage to the pathogen, we should also see if there is any protective advantage for the host. Although there is no direct evidence in this direction, recent studies point to the possibilities that *H. pylori *infection protects against childhood diarrohea, gastro-oesophagial reflux disease, oesophagial cancers and asthma [9-11]. Eradication of *H. pylori *by antibiotic therapy has shown augmented incidence of some of these diseases in different populations. Also, due to eradication, *H. pylori *is at steep decline in the west and has been rightly dubbed as an 'endangered species' in the stomach [30,31]. Do we need to save and conserve *H. pylori *as an important beneficial organism and a marker of human history; or should we eradicate it completely? Is eradication that simple? In most developing countries, it is not achievable because of rampant drug resistance among local strains. Also, even if eradicated using future effective drugs, re-colonization will be difficult to avoid due to poor water hygiene and frequent contamination. Apart from need for future functional studies to link *H. pylori *to human disease or to project it as a commensal or a mutualist, region-centered epidemiological studies may be required to ascertain need for eradication or otherwise for different populations and societies. As far as a biological level definition of this pathogen goes, it is possible to term prehistoric versions of it as a 'bacterial parasite' [32], as it was so, prior to its acquiring the virulence genes (again it needs to be proved whether *H. pylori *has been always benign prior to the emergence of *Homo sapiens*). The present day *H. pylori *should not be dubbed as a beneficial organism or a commensal but only after its 'disarmament' - meaning that its powerful virulence machinery makes the organism a 'classical pathogen' and nothing less than that.

## Competing interests

The authors declare that they have no specific competing interests related to this manuscript except that NA is the Co-Editor in Chief of the *Gut Pathogens *journal.

## Authors' contributions

NA developed the hypothesis, wrote the text and sketched Figure 1. ST participated in molecular genotyping studies on *H. pylori *and NN worked on functional characterization of plasticity region proteins.

All authors have read and approved the final manuscript.
